# Antioxidant or pro-oxidant and glutathione transferase P1-1 inhibiting activities for *Tamarindus indica* seeds and their cytotoxic effect on MCF-7 cancer cell line

**DOI:** 10.1186/s43141-020-00077-z

**Published:** 2020-11-19

**Authors:** R. A. Guneidy, A. M. Gad, E. R. Zaki, F. M. Ibrahim, A. Shokeer

**Affiliations:** 1 Molecular Biology Department, National Research Centre, Cairo, Dokki Egypt; 2 Medicinal and Aromatic Plants Research Department, National Research Centre, Cairo, Dokki Egypt

**Keywords:** Cancer, Glutathione transferase, Phenolic compounds, Antioxidant/pro-oxidant balance, Anticancer drugs, Herb-drug interaction

## Abstract

**Background:**

The multidrug resistance (MDR) of cancer cells is a major obstacle to cancer treatment. Glutathione S-transferase Pi (GSTP1-1) catalyzes the conjugation of glutathione with anticancer drugs and therefore reduces their efficacy. Phenolic compounds have the potential to inhibit GST P1-1 activity, which is a promising goal to overcome MDR and increase the efficacy of chemotherapy.

**Results:**

Three fractions (dichloromethane, ethyl acetate, and *n*-butanol) were prepared from *Tamarindus indica* seeds to determine their phenolic and flavonoid properties as well as their antioxidant/pro-oxidant properties. The *n*-butanol fraction displayed the highest levels of phenol ( 378 ± 11.7 mg gallic acid equivalent/g DW) and flavonoids (83 ± 6.0 mg rutin equivalent/g DW). Inhibiting effects on purified GSTP1-1 activity in human erythrocytes (eGST), placenta (pGST), and hGSTP1-1 have been studied. The *n*-butanol fraction was the most effective in inhibiting eGST, hGSTP1-1, and pGST with IC_50_ values of 3.0 ± 0.7, 4.85 ± 0.35, and 6.6 ± 1.2 μg/ml, respectively. Cellular toxicity was investigated for the *T. indica n*-butanol fraction on various human cancerous cell lines. The only ones affected were MCF-7 cell lines (72%) and HePG2 (52%) indicated cytotoxicity. The value of IC_50_ is 68.5 μg/ml of *T. indica n*-butanol fraction was observed compared to 1.7 μg/ml tamoxifen in MCF-7 cell lines. The combination of treatment of *T. indica* extract with the medicinally approved drug tamoxifen had unexpected effects; complete elimination of the cytotoxic inhibition effect of tamoxifen and the plant extract was observed.

**Conclusions:**

However *T. indica* extract has a cytotoxic effect on the MCF-7 cell line; in certain situations, plant products can have an opposite effect to the intended drug, which decreases the impact of the drug.

## Background

Cancer is a complex disease with multiple factors, such as genetics; epigenetics, environment, and lifestyle interact strongly during the cancer process. Major cancer treatment strategies include chemotherapy, radiotherapy, and surgery, and in some cases, joint strategies have shown the best results for improving cancer survival. However, due to the high heterogeneity of cancer cells, it is extremely difficult to predict which cells will respond to treatment, indicating that resistance to treatment is a major problem for patients [1].

Multidrug resistance (MDR) for cancer cells limits the therapeutic effect of anticancer drugs and is a major barrier to cancer treatment. Multidrug-resistant cells involve complex steps that target different metabolic pathways for tumor development. One of the most classic MDR resistance mechanisms is increased levels of detoxification enzymes, changes in drug uptake and flow, and deactivation of apoptosis pathways [2, 3] .

Glutathione transferase (GST) is a family of enzymes that catalyze the glutathione (GSH) -dependent detoxification of various chemotherapeutic drugs to reduce their cytotoxic effects. GST catalyzes the conjugation of GSH with anticancer drugs to prevent direct drug activity; this conjugation reduces the ability of anticancer drugs to penetrate the cell and to attack cellular DNA [4]. This activity appears to occur preferably via isoform (GSTP1-1); GSTP actually interacts with a number of small molecules and cellular proteins that produce regulatory effects across the main signaling and transcription pathways (identified as the “GSTP regulatory interactome”). Many human cancers have been shown to express high levels of GSTP1-1 and their expression has been correlated with both disease progression and chemotherapy resistance. Drugs targeting GSTs are tested in clinical trials as adjuvant therapies with anticancer agents to enhance the effectiveness of chemotherapy. Inhibiting GST activity can inhibit the function of the C-Jun N-terminal kinase (JNK) signal regulator and thus improving cell signaling pathways in cancer cells and apoptosis [5, 6].

Polyphenols of plant origin or their synthetic derivatives have been identified as redox-active molecules with relatively low toxicity. They have a wide range of beneficial effects, including anti-inflammatory, anti-mutagenic, and anti-carcinogenic actions [7–9].

Phenolic compounds have been categorized by source, biological function, and chemical structure. They can be subdivided into two main classes, flavonoid and non-flavonoid (for example, phenolic acids, phenolic alcohols, and tannins) based on the number of aromatic rings and their binding affinity to different compounds [10]. Flavonoids may be divided into different subclasses depending on the degree of oxidation of the heterocyclic ring as: anthocyanins, flavonols, flavans, flavanol, flavones, and isoflavones [11]. Phenolic and flavonoid compounds have the potential to modulate the activity and/or expression of GSTs. This impact on GSTs depends heavily on the composition, concentration, length of administration, as well as on GST isoform and origin [12]. Some phenolic compounds have the ability to inhibit GST activity such as chlorogenic acid, ellagic acid, quercetin, curcumin, tannins, and stilbene, which has been seen as a promising target to overcome MDR and increase the efficacy of chemotherapy [13].

Phenolic compounds may have a pro-oxidant and cytotoxic effect. The pro-oxidant properties of such compounds depend on a variety of variables, including the phenolic class, the presence of oxygen or transition metal ions, alkaline pH, their concentration, and solubility characteristics [14]. For example, in most studies conducted on phenolic pro-oxidant behavior, copper is considered to be a potent catalyst for oxidative reactions. Phenolic pro-oxidant activity can cause cellular lipid peroxidation, DNA damage, and apoptosis [15]*.*

The pro-oxidant potential of certain polyphenols (e.g., quercetin, epigallocatechin gallate) has been established as chemotherapeutic adjuvants as they selectively promote the cytotoxic effects of chemotherapy and play an important role in the prevention of some forms of cancer based on their ability to selectively destroy cancer cells without influencing normal cells [2, 15, 16].

*Tamarindus indica L*. (*T. indica*) is a tropical plant mainly used in food and confectionery. It is one of the world’s traditional herbal medicines. Plant seeds are often disposed of as waste material due to their fragile and tasteful properties; however, they are nutritionally abundant. Seed extracts are found to have potent antioxidant potential and anti-stress. While *T. indica* leaf extract had anti-apoptotic effects, its complete seed extracts derived from the seed kernel and derived from the coat have antioxidant, cytotoxic, and immune [17].

The main objective of this study was to determine the efficacy of *T. indica* extract on cancer inhibition either alone or with traditional chemotherapy dependent on the inhibition of human GSTP1-1 and oxidative status.

## Methods

### Chemicals

Solvents for the extractions, dichloromethane, ethyl acetate, ethanol, and *n*-butanol were purchased from Merck Company. Folin-Ciocalteau-phenol reagent (FC), reduced glutathione (GSH), and 1-chloro-2, 4-dinitrobenzene (CDNB) were purchased from Merck Company. Epoxy activated-Sepharose 6B, 1, 1 diphenyl-2-picryl-hydrazyl (DPPH), ascorbic acid, and xylenol orange were purchased from Sigma-Aldrich Company. Phenolic compounds including Apigenin-7-glucoside, catechin, cinnamic acid, caffeic acid, curcumin, chlorogenic acid, epigallocatechin gallate, ferulic acid, gallic acid, gentisic acid, 18-α glycrrytinic acid**,** hesperidin, quercetin, *p*-coumaric acid, syringic acid*,* protocatechuic acid*,* pyrogallol, and vanillic acid, were purchased from Sigma-Aldrich Company. All other chemicals were of the highest purity commercially available.

### Preparation of plant extracts

Dry *T. indica* seeds were purchased from different localities and markets in Egypt, at various periods, as the plant used in the study is a plant known in the local market with traditional uses. Dry *T. indica* seeds (250 g) washed with water, dried at room temperature for 6 days and ground into fine powder using a domestic blender, mixed with solvents (1:5 w/v) of increasing polarity: dichloromethane, ethyl acetate, and *n*-butanol. The mixture was allowed to stand at room temperature for 12 h in the dark, with occasional agitation. The partitioned fractions were centrifuged at 1000*×g* for 10 min, filtered through Whatman No. 1 filter paper, and evaporated to dryness. The obtained dry weights (DW) of the three fractions were weighted and saved at −4 °C for further analysis.

### Phytochemical analyses

#### Determination of total phenol content

The antioxidant property of phenolic compounds is based on their capacity to scavenge free radicals by Folin Ciocalteau reagent (FC), (3H_**2**_O-P_**2**_O_**5**_-13WO_**3**_-5MoO_**3**_-10H_**2**_O). The colorimetric method is based on a chemical reduction of the reagent which is a mixture of tungsten and molybdenum oxides. Total concentration of phenolic compounds in the partitioned fractions was determined using a series of gallic acid standard solutions (2.5‑20 μg/ml) as described by Singleton and Rossi [18]. Each extract solution (0.1 ml) and the standard solutions of gallic acid were mixed with 2 ml of a 2% (w/v) sodium carbonate solution and vortexed vigorously. After 3 min, 0.1 ml of Folin Ciocalteau’s phenol reagent was added and each mixture was vortexed again. After incubation for 30 min at room temperature, the absorbance at 750 nm of each mixture was measured. The total phenolic content of plant extract was represented as mg of gallic acid equivalent (GAE)/g seed using an equation derived from the standard gallic acid calibration curve.

#### Determination of total flavonoid content

Total concentration of flavonoid compounds in *T. indica* fractions was determined using a series of standard rutin solutions (2.5‑50 μg/ml) as described in the aluminum chloride colorimetric method [19]. Aluminum chloride (AlCl3) forms labile acid complexes with orthodihydroxyl groups in the flavonoid A- or B-ring. Aluminum ion reactions with flavonoid in the alkaline medium phase of red chelate showing maximum absorption at 510 nm. A known volume of each extract solution was mixed with 5% sodium nitrite solution, vortexed vigorously, then 10% aluminum chloride solution was added and vortexed again. After 6 min, 4.3% of sodium hydroxide solution was added, followed by addition of water and each mixture was vortexed again. At the end of incubation for 2 h at room temperature, the absorbance of each mixture was measured at 510 nm. Total flavonoid content was expressed as milligram rutin equivalent (mg rutin/g seed).

#### Determination of the antioxidant capacity using the free radical scavenging activity method

The 1, 1 diphenyl-2-picryl-hydrazyl (DPPH) free radical scavenging activity of each sample was determined according to the method described by Blois [20] and Leong and Shui [21]. The initial absorbance of the DPPH solution (0.1 mM) in absolute ethanol was measured and adjusted until the absorbance equal 1.3 at 517 nm and did not change throughout the period of assay. A series of extract solutions with varying concentrations were prepared, 0.1 ml of solutions from each extract was added to 1.4 ml of DPPH solution. The absorbance at 517 nm was recorded after 30 min of incubation at room temperature. IC_50_ concentrations were calculated after constructing the percent inhibition versus log extract concentrations curve.

#### Determination of the pro-oxidant capacity using hydrogen peroxide production activity

The ability to produce hydrogen peroxide (H_2_O_2_) by the three fractions of *T. indica* and some phenolic compounds were investigated using the ferrous ion oxidation–xylenol orange (FOX) assay [22, 23]. A known volume of the plant extracts (500 μl) and 1 mM of different phenolic compounds were incubated in 50 mM sodium phosphate buffer, pH 7.4 in a final volume of 2 ml at 37 °C for 24 h with shaking in the dark. After incubation, the samples (33.3 μl) were mixed with 1 ml of FOX reagent (250 μM FeSO_4_, 25 mM H_2_SO_4_, 100 μM xylenol orange, and 100 mM sorbitol) according to previously described FOX (ferrous ion oxidation xylenol orange) method [22]. The reaction mixture was vortexed for 5 s and then incubated at room temperature for 30 min. Solutions were centrifuged at 2000×*g* for 10 min, and the absorbance at 560 nm was measured against FOX reagent. The FOX assay was calibrated using a standard H_2_O_2_ solution to cover the range of 2‑20 μM.

#### Detection of phenolic compounds using high-performance liquid chromatography

HPLC analysis was carried out for the dichloromethane, ethyl acetate, and *n*-butanol fractions of using an Agilent Technologies 1100 series liquid chromatograph equipped with an auto sampler and a diode-array detector. The analytical column was eclipse XDB-C18 (150 × 4.6 μm; 5 μm) fitted with 4.0 × 3.0 mm i.d. guard column. The mobile phase consisted of acetonitrile (solvent A) and 2% acetic acid in water (v/v) (solvent B). The flow rate was 1.0 ml/min for a total run time of 70 min and the gradient program was as follows: 100% B to 85% B in 30 min, 85% B to 50% B in 20 min, 50% B to 0% B in 5 min, and 0% B to 100% B in 5 min. There was 10 min of post-run for reconditioning. Peaks were monitored simultaneously at 280, 320, and 360 nm. All samples were filtered through a 0.45-μm Acrodisc syringe filter (Gelman Laboratory, MI) before injection. Peaks were identified by congruent retention times and UV spectra and compared with those of the standards [23].

### Biological materials

#### Expression and preparation of recombinant hGSTP1-1

The wild-type human GSTP1-1 was kindly gifted from Prof. Bengt Mannervik (Stockholm University, Department of Biochemistry and Biophysics, Sweden). The hGSTP1-1 was expressed in *E. coli* strain XL1-Blue from the plasmid pKK-D. The cells were grown to an OD_600_ of 0.2 and induced with 0.2 mM isopropyl-β-D-thiogalactoside (IPTG). The culture was incubated for 18 h at 37 °C and the cells were harvested at 3000×*g* for 10 min. Bacteria were suspended in buffer A (20 mM Tris-HCl, pH 8.0, 1 mM EDTA, 0.2 mM DTT), 0.2 mg of lysozyme\ml for 30 min on ice. Sonication was performed in 3 × 60s treatments, with 60s intermittent periods. The resulting material was centrifuged at 30,000×*g* for 30 min at 4 °C and the supernatant (hGSTP1-1) was collected for purification.

#### Preparation of human erythrocyte homogenate

Venous blood (20 ml) was collected in ethylene diamine tetra acetic acid, sodium salt (EDTA) containing tube, and centrifuged within 1 h of sampling at 1030×*g* for 10 min. The plasma and buffy layer were then removed; the erythrocytes were washed three times with a 9.0 g/L NaCl solution, and hemolyzed by the addition of an equal volume of ice-cold distilled water to yield a 50% cytosolic hemolysate [24].

#### Preparation of human placenta homogenate

Human placenta (38–40 weeks gestation) (*n* = 3) obtained from healthy women with no known history of any physiological or pathological problems were used in this study. The placenta was collected immediately after delivery from Imbaba General Hospital (participants gave informed consent). Placental tissue (14 g) was dissected, placed in 50% (w/v) of ice-cold 25 mM Tris-HCl buffer, pH 8.0 containing 1 mM DTT, 5 mM EDTA, homogenized immediately, centrifuged at 10,000×*g* for 15 min and saved at −4 °C for further analyses [25].

#### Purification of GSTs by affinity chromatography

Cytosolic fractions of erythrocyte and placenta were purified by affinity chromatography using a GSH-Sepharose 6B column. Reduced GSH was coupled to Epoxy-activated Sepharose 6B according to Simons and Vander-Jagt [26]. GST containing fractions were applied to the GSH Sepharose column (1.4 × 16.5 cm i.d.) previously equilibrated with 25 mM Tris-HCl buffer pH 8.0 containing 1 mM DTT, 5 mM EDTA at a flow rate of 30 ml/h, and the column was subsequently washed with the same buffer until no absorbance of the effluent at 280 nm was observed. The bound GST was eluted with 50 mM Tris-HCl buffer, pH 9.6 containing 10 mM GSH at a flow rate of 15 ml/h. Fractions with 2 ml were collected. Column fractions were monitored for protein at 280 nm and for GST enzymatic activity at 340 nm.

The recombinant hGST P1-1 was purified by using Ni-immobilized metal affinity chromatography (Ni-IMAC) affinity chromatography in one-step purification. The matrix was equilibrated with 20 mM sodium phosphate, pH 7.4, 0.5 M NaCl, 0.2 mM DTT, 0.1 M imidazole (buffer A). The bound GST was eluted with buffer A containing 0.5 M imidazole and dialyzed against 10 mM Tris-HCl, pH 7.8, containing 1 mM EDTA, and 0.2 mM DTT. The homogeneity of the purified fractions was analyzed by native polyacrylamide gel electrophoresis (PAGE) (7%) as described by Davis [27].

#### GST activity determination

The activity of GST was determined by measuring the increase in the concentration of the conjugation product of GSH and CDNB at 340 nm over 3 min at 25 °C [28]. Unless otherwise stated, the assay mixture contained in a total volume of 1 ml, 0.1 M potassium phosphate buffer, pH 6.5, 1 mM CDNB in ethanol (final concentration of ethanol less than 4%), 1 mM GSH, and the enzyme solution. One unit is equivalent to the amount of enzyme conjugating 1 μmole of CDNB in 1 min at 25 °C. The extinction coefficient of the product was taken to be 9.6 mM^−1^ cm^−1^. Protein was estimated using Coomassie brilliant blue G-250 and bovine serum albumin as standard [29].

#### Inhibition studies

Under the standard assay conditions, the effect of *T. indica* fractions was tested for their ability to inhibit the GSH-conjugating activity of GST. The inhibitors IC_50_ values were determined by measuring the activity of the enzyme in the presence of varying concentrations of the inhibitor and plotting the percentage activity values versus log inhibitor concentration.

#### Kinetic studies

All kinetic and inhibition studies were carried out using the purified preparations of the enzyme in the presence and absence of the inhibitor at a concentration, which causes 50% inhibition of enzyme activity (IC_50_). The apparent *K*_M_ and *V*_max_ values for GSH were determined at pH 6.5 using a GSH range from 0.1 to 2 mM and a fixed CDNB concentration of 2.0 mM. The apparent *K*_|M_ and *V*_max_ values for CDNB were determined using a CDNB range from 0.625 to 2 mM at a fixed GSH concentration of 5.0 mM. Data were plotted as double reciprocal Lineweaver–Burk plots to determine the apparent *K*_M_ values.

### Cytotoxicity and cell viability assay

#### Cell line authentication

The following human cell lines were identified and used: hepatocellular carcinoma cell line, **HePG 2**; colon cell line, **HCT116**; lung carcinoma cell line, **A54**9; breast cancer cell, **MCF-7**; prostate cell line, **PC3** and the normal skin fibroblast, **BJ1**.

#### Cell culture

All the following procedures were done in a sterile area using a Laminar flow cabinet biosafety class II level (Baker, SG403INT, Sanford, ME, USA). Cells were suspended in DMEM medium HCT116, 1% antibiotic-antimycotic mixture (10,000 U/ml potassium penicillin, 10,000 μg/ml streptomycin sulfate, and 25 μg/ml amphotericin B), and 1% l-glutamine at 37 °C under 5% CO_2_. Cells were batch cultured for 10 days, then seeded at a concentration of 10 × 10^3^ cells/well in fresh complete growth medium in 96-well microtiter plastic plates at 37 °C for 24 h under 5% CO_2_ using a water-jacketed carbon dioxide incubator (Sheldon, TC2323, Cornelius, OR, USA). Media was aspirated; fresh medium (without serum) was added and cells were incubated either alone (negative control) or with different concentrations of sample (extract) to give a final concentration of 100-50-25-12.5-6.25-3.125-1.56 and 0.78 μg/ml.

#### Cell viability assay

Cell viability was assessed by the mitochondrial-dependent reduction of yellow MTT (3-(4, 5-dimethylthiazol-2-yl)-2, 5-diphenyl tetrazolium bromide) to purple formazan [30]. After 48 h of incubation, the medium was aspirated, 40ul MTT salt (2.5 μg/ml) was added to each well and incubated for further 4 h at 37 °C under 5% CO_2_. To stop the reaction and dissolving the formed crystals, 200 μL of 10% sodium dodecyl sulfate (SDS) in deionized water was added to each well and incubated overnight at 37 °C [31]. The absorbance was then measured using a microplate multi-well reader (Bio-Rad Laboratories Inc., model 3350, Hercules, California, USA) at 595 nm and a reference wavelength of 620 nm. DMSO is the vehicle used for the dissolution of plant extracts and its final concentration on the cells was less than 0.2%. The percentage of change in viability was calculated according to the formula:

((Reading of extract/reading of negative control)−1) × 100

### Statistical analysis

All data are reported as mean ± SD for *n* = 3–4 independent experiments. The Student’s *t* test was conducted to analyze the disparity between the means. The *p* values of less than 0.05 were considered to be significant. For cell viability assay, statistical significance was tested between samples and negative control (vehicle cells) using an SPSS 11 independent *t* test. A probit analysis for IC_50_ and IC_90_ was carried out using the SPSS 11 system.

## Results

### Bioactive compounds for the three *T. indica* partitioned fractions

#### Total phenolic and flavonoid contents

The total phenols and flavonoids of *T. indica* partitioned fractions: dichloromethane, ethyl acetate and *n*-butanol are summarized in Table 1. The three fractions have a different extraction yields and obtained dry weights (DW) of 5, 9.84, and  30.9 g, respectively. The *n*-butanol fraction showed the highest phenolic contents ( 378± 11.7 mg GAE/g DW), followed by the ethyl acetate fraction (122 ± 1.6 mg GAE/g DW, *p* < 0.001) and the dichloromethane fraction (49 ± 1.6 mg GAE/g DW, *p* < 0.001). High flavonoids were also observed for fraction of *n*-butanol (83 ± 6 mg RE/g DW) followed by a fraction of dichloromethane (31 ± 4 mg RE/g DW) and ethyl acetate fraction (23 ± 2 mg RE/g DW, *p* < 0.001). The ratio of flavonoids to total phenols in the dichloromethane fraction was high (0.64), while the same ratio was observed in ethyl acetate (0.19) and *n*-butanol fractions (0.22).

**Table 1:** Total phenol (TPC) and flavonoid (TFC) contents, antioxidant capacity (IC_50_ of DPPH scavenging activity), and hydrogen peroxide production activity for *T. indica* fractions

Fractions	Extraction yield (g DW)	TPC (mg GAE. gDW^**−1**^)	TFC (mg RE. gDW^**−1**^)	F/P	IC_**50**_ (mg.gDW^**−1**^)	μmol H_**2**_O_**2**_ (g DW^**−1**^)
**Dichloromethane**	5.0	49 ± 1.6	31 ± 4.0	0.64	165 ± 11	8.7± 0.27
**Ethyl acetate**	9.84	122 ± 1.6	23 ± 2.0	0.19	45 ± 3.0	29.2 ± 2.9
***n*-butanol**	30.9	378 ± 11.7	83 ± 6.0	0.22	2.1 ± 0.08	77.5 ± 4.6

TPC (total phenolic content) was expressed as milligram of gallic acid equivalent per gram of extract dry weight (mg GAE. gDW^−1^)

TFC (total flavonoid content) was expressed as milligram of rutin equivalent per gram of extract dry weight (mg RE. gDW^−1^)

*F/P* flavonoid/phenol ratio

The values represent the mean ± SD of three independent experiments

*Highly significant value *p*< 0.001

#### Antioxidant and pro-oxidant capacity of *T. indica* partitioned fractions

Results of Table 1 illustrate the dual nature of the antioxidant/pro-oxidant capacities of the three *T. indica* fractions. The IC_50_ values using DPPH scavenging activity showed that *n*-butanol fraction of *T. indica* seeds has a powerful antioxidant capacity ( 2.1± 0.08 mg/g DW, *p* < 0.001) followed by the ethyl acetate fraction (45 ± 3 mg/g DW) and the dichloromethane fraction (165 ± 11 mg/g DW). The production of H_2_O_2_ was monitored by the *T. indica* partitioned fractions after incubation for 24 h. Fraction of *n*-butanol showed the highest production capacity of H_2_O_2_ ( 77.5 ± 4.6 μmol/mg DW of extract, *p* < 0.001) followed by the ethyl acetate fraction (29.2 ± 2.9 μmol/mg DW) and the dichloromethane fraction (8.7 ± 0.27 μmol/mg DW).

#### Phenolic compounds of *T. indica* fractions using high-performance liquid chromatography

HPLC analysis was performed to identify and measure phenolic compounds extracted from the three *T. indica* fractions. Eight phenolic compounds were identified in the three fractions as shown in Table 2. Identification of phenolic compounds in the three *T. indica* fractions by HPLC analyses showed the presence of protocatechuic acid at the highest concentration in the *n*-butanol fraction (307.5 μg/g DW) followed by the fraction of ethyl acetate (146.7 μg/g DW) and dichloromethane fraction ( 119.69 g/g DW). This main phenolic acid accounts for 42.75%, 40.52%, and 39.5% of the total phenols detected in dichloromethane, ethyl acetate, and *n*-butanol fractions, respectively. Large amounts of *p*-hydroxybenzoic (78.34 μg/g DW) were observed in dichloromethane fraction at 28% of total phenols. Small amounts of *p*-hydroxybenzoic (7.80 μg/g DW) were also present in the ethyl acetate fraction at 2.15%, while not detected in the *n*-butanol fraction. Large amounts of syringic acid (225.7 μg/ g DW) were observed in the *n*-butanol fraction and in the ethyl acetate fraction (71.5 μg/g DW) at 29% and 19.75%, respectively, of total phenols. The presence of vanillic acid, *p*-coumaric acid, and caffeic acid in the three *T. indica* fractions was less than 5% of total identified compounds. Rutin of the flavonoids was observed only in the ethyl acetate fraction (20.1 μg/g DW) at 5.55%. A large amount of non-flavonoid catechin is present in the *n*-butanol fraction (134 μg/g DW, 17.2%) followed by the ethyl acetate fraction (97.7 μg/g DW, 27%) and dichloromethane fraction (61.86 μg/g DW, 22.1%), Table 3.

**Table 2:**
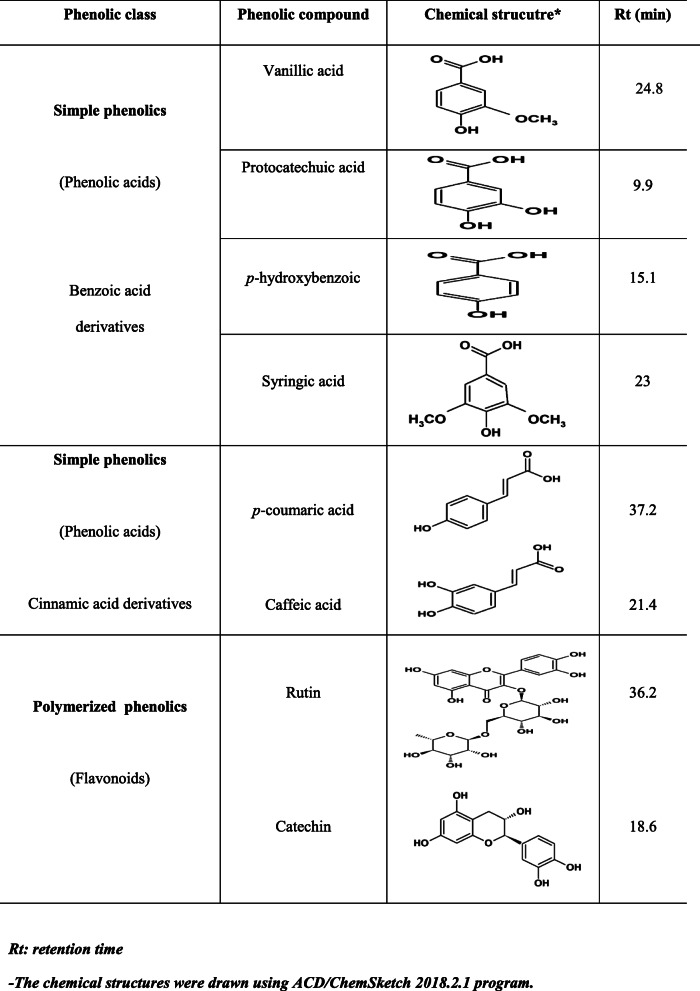
The identified phenolic compounds in the three *T. indica* fractions using HPLC

**Table 3:** Quantitation of the identified phenolic compounds in the three *T. indica* fractions

Phenolic compound type	Identified compounds	***T. indica*** fractions					
		Dichloromethane (μg/g DW)	% (w/w)	Ethyl acetate (μg/ g DW)	%	***n***-butanol (μg/ g DW)	%
**Phenolic acids**	**Vanillic acid**	20.01	7.15	12.5	3.45	96.6	12.4
	**Protocatechuic acid**	119.69	42.75	146.7	40.52	307.5	39.5
	***p*-hydroxybenzoic**	78.34	28	7.80	2.15	ND	
	**Syringic acid**	ND		71.5	19.75	225.7	29
	***p*-coumaric acid**	ND		5.70	1.57	ND	
	**Caffeic acid**	ND		ND		15.0	1.93
**Flavonoid**	**Rutin**	ND		20.1	5.55	ND	
	**Catechin**	61.86	22.1	97.7	27	134	17.2
	**Total phenolics**	280	100	362	100	779	100

*% (w/w)* % calculated relative to the total concentration of identified phenolic compounds, *ND* not detected

#### Inhibitory effect of *T. indica* fractions on purified GST activity

*Tamarindus indica* fractions were examined for their inhibitory effects on the predominant GST activity purified from human erythrocytes (eGST), placenta (pGST), and hGSTP1-1. The inhibitor concentration needed to achieve approximately 50% inhibition of GST activity, known as IC_50_, was calculated by plotting the relative values of residual activity versus extraction concentrations.

Regarding the amount of dry weight of the plant, the inhibitory effect results (IC_50_ values in μg/ml) shown in Table 4 showed that the *n*-butanol fraction was the most effective extract in inhibiting eGST, hGSTP1-1, and pGST with IC_50_ values 3 ± 0.7, 4.85 ± 0.35, and 6.6 ± 1.2 μg/ml, respectively. The ethyl acetate fraction has moderate inhibitory effects on the activities of eGST, pGST, and hGSTP1-1 with IC_50_ values of 27 ± 1, 56.66 ± 16.5, and 58 ± 15 μg/ml, respectively. Whereas dichloromethane extract has small inhibitory effects (IC_50_ = 64.54 ± 5.85, 93.74 ± 4.3 and 105.4 ± 3.3 for eGST, hGSTP1-1, and pGST) on the three studied GST activities. Calculating the percentage of phenol and flavonoids in IC_50_ inhibition values showed that the amount of phenol (≤ 37.6 μg GAE/g DW) and flavonoids (≤ 8.3 μg RE/g DW) in the *n*-butanol fraction that caused the inhibition effect more than their quantities in dichloromethane and ethyl acetate fractions (Table 4).

**Table 4:** IC_50_ values and phenol content for the *T. indica* fractions ability to inhibit the activity of purified GSTs

Extract of ***T. indica***	IC_**50**_ value	Erythrocyte GST	Placenta GST	hGSTP1-1
**Dichloromethane**	**Concentration** (μg/ml)	**64.54 ± 5.85**	**105.4 ± 3.3**	**93.74 ± 4.3**
	**Phenol** (μg GAE/g DW)	**3.15 ± 0.28 (4.9%)**	**5.14 ± 0.16 (4.9%)**	**4.6 ± 0.2 (4.9%)**
	**Flavonoid** (mg RE/g DW)	**2 ± 0.18 (3.1%)**	**3.2 ± 0.1 (3%)**	**2.9 ± 0.006 (3.1%)**
**Ethyl acetate**	**Concentration** (μg/ml)	**27 ± 1**	**56.66 ± 16.5**	**58 ± 15**
	**Phenol** (μg GAE/g DW)	**3.3 ± 0.12 (12.2%)**	**6.9 ± 2 (12.2%)**	**7.08 ± 1.8 (12.2)**
	**Flavonoid** (mg RE/g DW)	**1.3 ± 0.023 (4.8%)**	**1.3 ± 0.37 (2.3%)**	**1.33 ± 0.35 (2.3%)**
***n*-butanol**	**Concentration** (μg/ ml)	**3 ± 0.7**	**6.6 ± 1.2**	**4.85 ± 0.35**
	**Phenol** (μg GAE/g DW)	**1.13 ± 0.26 (37.6%)**	**2.5 ± 0.45 (37.9%)**	**1.83 ± 0.13 (37.7%)**
	**Flavonoid** (mg RE/g DW)	**0.25 ± 0.05 (8.3%)**	**0.55 ± 0.09 (8.3%)**	**0.4 ± 0.03 (8.2)**

IC_50_: amount of extract which causes 50% inhibition of enzyme activity

μg GAE/g DW: μg of gallic acid equivalent per gram of extract dry weight

μg RE/g DW: μg of rutin equivalent per gram of extract dry weight

The values represent the mean ± SD of three independent experiments

The values in parentheses represent the percentage relative to concentration in μg/ml

According to the current HPLC analysis of the three *T. indica* fractions and previous analysis on ethanol extracts (data not shown), twenty of the fixed phenol compounds were examined to estimate antioxidant/pro-oxidant potential and GSTP1-1 inhibition activity.

#### Effect of inhibitor on the kinetics of hGSTP1-1 activity

**Fig. 1:**
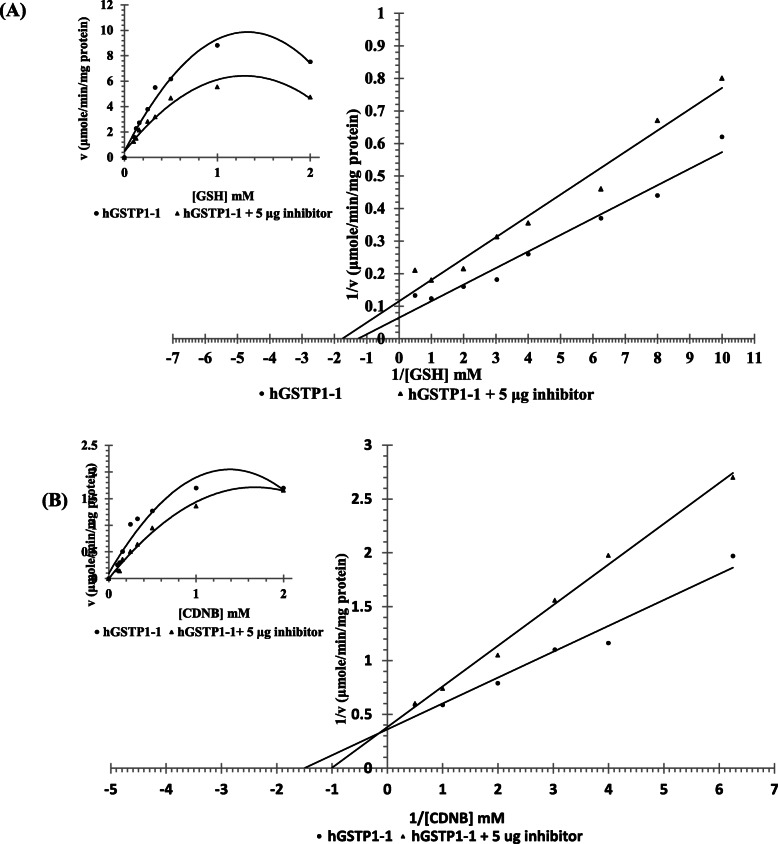
**a** Effect of GSH concentration variation on the activity of hGSTP1-1 in the presence and absence of 5 μg of *T. indica n*-butanol fraction. **b** Effect of CDNB concentration variation on the activity of hGSTP1-1 in the presence and absence of 5 μg of *T. indica n*-butanol fraction. Both Michaelis-Menten and Lineweaver–Burk plot was shown of two substrates. The GSH concentration was varied between 0.1‑2 mM at fixed CDNB concentration (2 mM) in the presence and absence of 5 μg of *T. indica* fraction (**a**). The CDNB concentration was varied between 0.625‑2 mM at constant GSH concentration (5 mM) in the presence and absence of 5 μg of *T. indica* fraction (**b**)

The most potent inhibitory *n*-butanol extract was chosen to determine the mechanism of enzyme inhibition (i.e., GSTP1-1). The effect of the concentration of GSH between 0.1‑2 mM on GSTP1-1 was tested at a fixed 2 mM of CDNB (Fig. 1a). The enzyme exhibited typical Michaelian activity in this range of GSH concentration, i.e., a linear relationship was observed when 1/v was plotted against 1/[S], Fig. 1a. The *K*_M_ value was calculated to be 0.83 mM with a *V*_max_ equal 16.7 μmoles/min/mg protein. Addition of 5 μg of *T. indica* fraction inhibits the enzyme activity with an observed decrease in the *K*_M_ value to 0.56 mM, and *V*_max_ value to 8.3 μmoles/min/mg protein (Fig. 1a).

The effect of CDNB concentration in the range between 0.625 and 2.0 mM on GSTP1-1 activity was examined at a fixed GSH concentration of 5 mM. The enzyme exhibited a typical Michaelian behavior in this range of CDNB concentration (Fig. 1b). The *K*_M_ value was determined to be 0.66 mM with a *V*_max_ equal 2.5 μmoles/min/mg protein. GST activity was assayed with variable concentrations of CDNB (0.625‑2 mM) in the presence of 5 μg of the *T. indica* fraction at GSH concentration of 5 mM. Lineweaver–double reciprocal plot showed that the value of *V*_max_ remained unchanged (2.5 μmoles/min/mg protein) with a *K*_M_ value of 0.66 mM increased to 1 mM for CDNB (Fig. 1b). The observed behavior of *T. indica* extract indicated that it inhibits GSTP1-1 activity with respect to GSH in an uncompetitive manner. In fact, competitive inhibition type was found with respect to CDNB.

#### The cytotoxic effect of *T. indica* extract on different human cancer cell lines

**Fig. 2:**
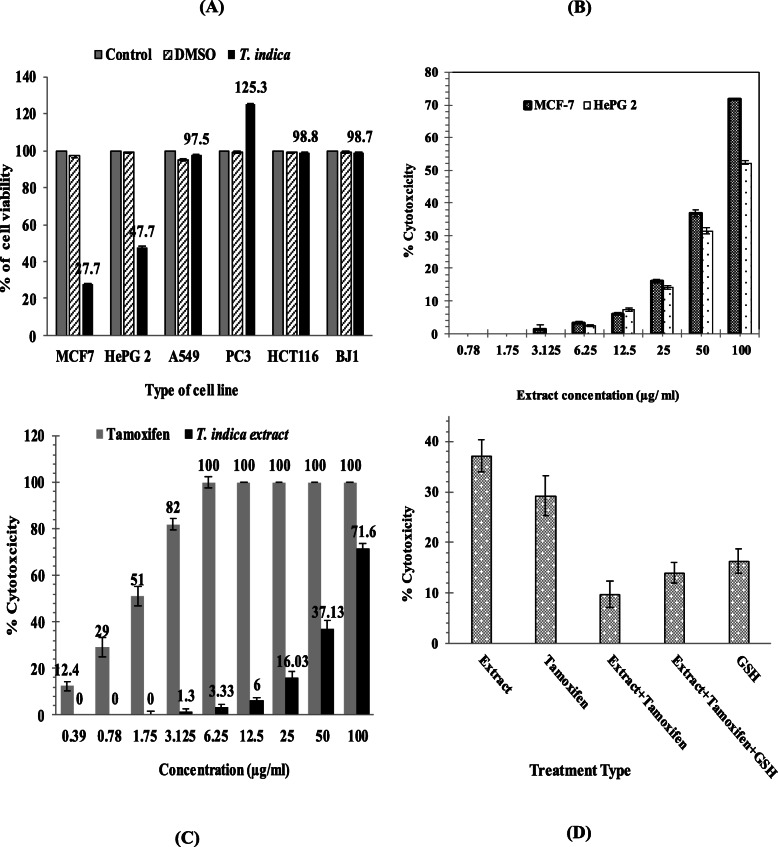
Cytotoxic effect of *T. indica* fraction (**a**) on different human cancer cell lines including: liver HePG2, colon HCT116, lung A549, breast MCF-7, prostate PC3, and the normal skin fibroblast, BJ1; (**b**) using different concentrations on human breast MCF-7 and hepatocellular carcinoma, HePG2; (**c**) using different concentrations of extract and tamoxifen on MCF-7 cell count and viability (**d**) combination treatment of IC_25_ concentration of *T. indica* fraction (47.3 μg/ml); tamoxifen (0.8 μg/ml), glutathione (GSH) on MCF-7 cytotoxicity. Error bars represent standard deviation from the mean (┬)

Cytotoxic activity for *n*-butanol fraction of *T. indica* on various cancer cell lines, including HePG2, HCT116, A549, MCF-7, and PC3, as well as normal skin fibroblast, BJ1; shown in Fig. 2a. Extract cytotoxicity was examined in the range of 0.78 to 100 μg/ml compared to untreated or DMSO-treated cells as a negative control. Dose-dependent inhibition of *T. indica* extract was observed in MCF-7 with 72% (*p <* 0.05) inhibition of cellular viability at an IC_50_ value of 68.5 μg/ml (Fig. 2b) compared to 26.1 μg/ml of doxorubicin (as positive control) and 1.7 μg/ml of tamoxifen (Fig. 2c), respectively. Extract of *T. indica* has a cytotoxic effect on the HePG2 cell line after cells have been incubated for 48 h, raising the concentration from 0.78 to 100 μg/ml of extract with 52% inhibition of cell viability (*p <* 0.05).

As shown in Fig. 2a, the cell viability of normal skin fibroblast, BJ1, and other cancer cell lines, i.e., colon HCT116 and lung A549, were not affected by *T. indica* extract. However, an increase in the number of cells in the prostate cancer PC3 cell line was reported, with an increase in cell viability of a percentage to 125.3.

#### Combination treatment

Tamoxifen-*T. indica* fraction combined treatment was measured using their IC_25_ concentrations. The effect of reduced glutathione (GSH) on the cytotoxicity (Tamoxifen-*T. indica*-GSH) of the MCF-7 cell line relative to untreated cells was also examined. The results shown in Fig. 2d showed that the combination of 47.3 μg/ml of extract (37.13% of cytotoxicity) and 0.8 μg/ml of tamoxifen (29.3% of cytotoxicity) significantly decreased from the cytotoxicity of the MCF-7 cell line to 9.7% (*P* ˂ 0.001). The addition of GSH insignificantly raised the risk of cytotoxicity to 14% for the treatment of tamoxifen-*T. indica-*GSH (*P* ˃ 0.05), but GSH alone showed 16.3% of MCF cytotoxicity.

## Discussion

Biologically active natural compounds have the ability to modify many intracellular signals including apoptosis, proliferation, differentiation, migration, metabolism, and much more. Extracts from natural sources are typically multi-target, less toxic, abundant, economical, and a mixture of a bioactive group of compounds [17]. Some medicinal plants and their bioactive constituent compounds that exert anticancer properties act against several molecules such as growth factors, transcription factors, kinases, enzymes, cytokines leading to inhibition of cell proliferation, induction of apoptosis, and cell death. Examples include *Curcumin longa* (turmeric), *Allium sativum* (garlic), *Camellia sinensis* (green tea), *Punica granatum* (pomegranate), *Panax ginseng* (ginseng), *Rhus verniciflua* (Chinese lacquer tree), and several of the constituent compounds, i.e., curcumin, resveratrol, genistein, gingerols, and quercetin, which may be incorporated into clinical practice [32, 33].

Plants contain a number of chemicals, including phenolic acids, which have been identified as potential internal biological activity, and methods of extraction are commonly used for their recovery. Growing plant produces and accumulates a certain amount of these compounds in its tissues, and their attractiveness to solvents varies by polarity [34]. The first step in the use of phytochemicals is the extraction of bioactive compounds from plant materials. Phenolic solubility depends on the chemical composition and physical properties of the plant sample, the polarity of the solvents used, the time of extraction, the temperature, and the solvent ratio of the sample. The phenolic composition of plants ranges from simple (e.g., phenolic acids) to strongly polymerized compounds (e.g., tannins) in various amounts. Solvents, e.g., methanol, ethanol, acetone, ethyl acetate, often with varying amounts of water, have been used to extract plant phenolic compounds [35]. The difference in the type of solvent could be due to the characteristics of each organic solvent that extracted only certain selective components and the ability of these solvents to dissolve compounds together with phenols [36].

In this fractionation analysis, three fractions (dichloromethane, ethyl acetate, and *n*-butanol) were partitioned from *T. indica* seeds to test their phenolic and flavonoid contents along with their antioxidant/pro-oxidant capabilities. Table 1 shows the dry weight produced, the phenol and flavonoid contents combined with the antioxidant/pro-oxidant potential in each fraction, showing that the *n*-butanol fractions displayed the highest values for these measured parameters. Dichloromethane and ethyl acetate fractions, on the other hand, showed poor recovery yields with the same measured parameters. Nakamura et al. [34] showed that highly polarized solvents such as aqueous and *n*-butanol fractions have been shown to generate higher extraction yields and total phenol content of ethyl acetate fractions of bamboo leaves. These results are consistent with the results of our study. However, higher extraction yields were produced in another study on the extract of orange peel with ethyl acetate. The observed variation between the results in the literature can be explained, since the production of extracted materials is significantly affected not only by the polarity of the solvent but also by other factors, including the types of plant parts, storage times, and temperature.

In general, low-polar molecules tend to dissolve more easily using low-polar solvents [34]. According to the results in Table 1, the ratio of flavonoids to phenol contents of the three fractions *T. indica* showed that the solubility of flavonoids was high in the fraction of dichloromethane (ratio = 0.64) whereas the same ratio was observed in the fractions of ethyl acetate and *n*-butanol (ratio = 0.19 and 0.22, respectively). These findings suggest that simple phenolic compounds at high concentrations may be present in *n*-butanol *T. indica* fraction and thus contribute to their high antioxidant/pro-oxidant potential.

Our results clearly demonstrate the relationship between the level of phenolic and flavonoid compounds in plant extracts and their antioxidant capacity. *Tamarindus indica n*-butanol fraction having the greatest volume of phenolic compounds, also has the highest antioxidant capacity (Table 1).

Several studies reported that the antioxidant capacity is directly correlated with phenol compounds and flavonoids [9, 37, 38]. The observed antioxidant effect of these extracts can be due to their electron donation capacity, thus forming a stable substance and consequently terminating a free radical chain reaction. The high scavenging properties of the extracts can be attributed to their structural conformation. Although phenolic containing high concentrations of hydroxyl groups can reduce DPPH particles very quickly, it can provide the required component as a radical scavenger [39]. In our experimental conditions, *n*- butanol fraction of *T. indica* has a good ability to act as antioxidant and pro-oxidant.

In certain cases, phenolic compounds were known to be anti-inflammatory, toxic, and mutagenic compounds. The toxicity of phenolic compounds has not been completely identified and has been overlooked for several years [14]. Phenolic compounds can have a pro-oxidant effect under some conditions. The pro-oxidant activity of a number of phenolic compounds that induce oxidative stress, either by the formation of reactive oxygen species (ROS) or by inhibiting antioxidant systems, resulting in oxidative damage to biological macromolecules such as lipids, proteins, and DNA resulting in cellular damage. This pro-oxidant action, unlike its antioxidant properties, is not necessarily harmful to biological systems and human health and can be used therapeutically to treat oxidative stress and as a possible anticancer mechanism [15, 40].

GST inhibitors may increase the sensitivity of cancer cells to chemotherapeutic agents and may therefore be used in a variety of therapeutic applications. For this reason, a large number of GST inhibitors have been synthesized with good specificities and reduced toxicity. Many natural inhibitors present in plants were also described and studied [41].

A large number of plant bioactive compounds with anti-oxidant, anti-mutagenic, and anti-carcinogenic properties have gained attention. Plant phenolic and polyphenolic compounds that play an important role in human nutrition are among these bioactive compounds [42]. Some plant extracts, such as tea components (epigallocatechin gallate) are classified as cytochrome-P450 (CYP) inhibitors of drug metabolism disorders. Fruit juices (e.g., grapefruit juice) may have a major effect on the pharmacokinetics and toxicity of drugs, as they can inhibit the action of certain enzymes involved in the metabolism of drugs [43, 44].

In the present study, the three *T. indica* fractions have been tested for their inhibition effects on the activity of the purified GSTs. Our results showed that *n*-butanol extract had the most effect on the GST activity of erythrocytes, placenta, and hGSTP1-1. The *T. indica* fraction is considered to have the highest overall phenolic and flavonoid content as a phenolic rich extract.

In particular, the present inhibition activity of the three *T. indica* fractions indicated that GST of erythrocyte was the most inhibited enzyme than placenta GST and hGST P1-1. This sensitivity may be due to genetic variants.

In the human genome, several genes located in class-specific clusters of different chromosomes are represented and expressed with unique patterns in each tissue. Many of these genes are polymorphic, resulting in variations of the GST phenotype. Genetic polymorphisms are hereditary alterations in DNA sequences such as single nucleotide polymorphisms or stable point mutations that may recur. Genetic polymorphisms are genetic variations in DNA sequences such as single nucleotide polymorphisms or stable point mutations that can result in a null phenotype. The main polymorphic GST genes are those coding for *GSTT1*, *GSTM1*, and *GSTP1* [41]. Among human populations, there is a significant degree of variation in the toxicity response. The GST variants have the potential to influence the response of various xenobiotic substances, drugs, and environmental stress. For example, CYP1A1 levels are known to differ 20–130 times between individuals, while 21-fold inter-individual variations in CYP1A1 enzyme activity have been recorded [45].

In the present study, IC_50_ inhibition values showed that the amount of phenol and flavonoids in the *n*-butanol fraction that caused the inhibition was greater than their quantities in dichloromethane and ethyl acetate fractions (Table 4).

The ability of phenolic agents to modulate other enzymes, such as hydrolases, transferases, kinase, oxidase, GST, cytochrome-P450, ATPase, lipase, and phospholipase, has been verified [46]. Plant extracts with high polyphenols are known to have a significant inhibitory effect on GST. Evidence shows that the mechanism by which plant polyphenols exercise defense against cancer and many diseases is not simply due to their antioxidant properties, but rather to their binding capacity to target proteins (or peptides). Such a mechanism of action that regulates various cell functions, i.e., induce inhibition of key enzymes, growth and proliferation, inflammation, apoptosis, and immune responses [32].

Our findings indicate the impact of *T. indica* inhibition is primarily due to higher total phenol concentration and can be associated with the presence of other mixtures of phenolic compounds. Therefore, an HPLC study was carried out to classify and quantify the phenolic compounds derived from the three *T. indica* fractions.

Protocatechuic acid was the highest concentration in the three fractions according to current results. Confirmed amounts of *p*-hydroxybenzoic acid and syringic acid were present among phenolic acids. Confirmed quantities of rutin and catechin have been detected among flavonoids. The chemical structure of the plant identified phenolics in the current study was listed in Table 2, as paradigm of the key structural features including (1) 3′, 4′-dihydroxy on ring B (e.g., catechin, quercetin), (2) double bond between carbon 2 and carbon 3 in the C ring, conjugated with the keto group at position 4, these allows conjunction between the ring A and B, or electron delocalization. (3) Presence of hydroxyl group substituents at position 3 of ring C and position 5 of ring A.

GSTs have affinity to a wide variety of chemical structures. Many compounds can act as inhibitors of GST activity by interacting with its substrates [47]. A variety of research findings have shown that phenols with various hydrophobic levels, such as flavonoids [48], cinnamic acid, coumaric acid [49], exhibited major interactions with GSTs. Other researchers have documented inhibitory effects of certain naturally occurring plant polyphenols on GST activity [50, 51]. The role of the GST is to catalyze the conjugation of the GSH sulfur atom to the electrophilic center of the endogenous and exogenous toxic compounds, increasing their solubility and excretion [52].

Most of the GSTs are made up of two identical subunits. The complete active site of each subunit consists of one binding site for GSH (G site) and one binding site for a number of hydrophobic substrates (H site) adjacent to the G site [53].

This research focuses on the mechanism of enzyme inhibition (i.e., hGST P1-1) with respect to the CDNB hydrophobic substrate, because inhibition of binding to this hydrophobic site (H site) changes the mode of interaction with the target protein (i.e., GST P1-1) and greatly reduces its spontaneous reactivity to GSH (G site). To assess whether inhibition of GSTP1-1 by the most potent inhibitory *n*-butanol extract of *T. indica* was competitive or non-competitive, Michaelis-Menten and Lineweaver-Burk plots were drawn for the non-inhibited and partly inhibited enzyme.

Kinetic studies with purified GST P1-1 show differences in the specific activity of GSH and CDNB substrates, the value of *K*_M_ may be increased or decreased depending on the type of GST isoform and the nature of the inhibitor. *K*_M_ values for GSH described seem to be lower than 1 mM. However, *K*_M_ values of CDNB differ 100 or more fold depending on the GST isoform. It should be remembered that the comparative values of these kinetic constants are difficult to obtain since the test conditions used by the investigators are not the same [54, 55].

The *K*_M_^GSH^ value of the enzyme and the reaction *V*_max_ value also decreased with the addition of *T. indica* extract to the reaction medium, suggesting the type of uncompetitive inhibition. The *K*_M_^CDNB^ value of the enzyme increased with the unaffected *V*_max_ value with the addition of *T. indica* extract to the reaction medium, suggesting the type of competitive inhibition. The *K*_M_^CDNB^ value for hGST P1-1 was 0.66 mM compared to the purified erythrocyte enzyme 0.78 mM 0.7 mM [54].

Competitive inhibitors interact with substrates to bind to the enzyme simultaneously. The inhibitor has an affinity to the active site of the enzyme, to which the substrate also binds. This form of inhibition can be overcome by increasing the concentration of the substrate, which is out competing the inhibitor. However, uncompetitive inhibitors bind to the enzyme at the same time as the substrate of the enzyme. However, the binding of the inhibitor affects the binding of the substrate and vice versa. Such form of inhibition cannot be reversed, but can be decreased by increasing the concentration of the substrate. The inhibitor typically has an allosteric effect where it binds to an enzyme site other than the substrate. This binding to the allosteric site shifts the conformation of the enzyme to decrease the affinity of the substrate to the active site.

The catalytic efficacy of the reaction decreased as the concentration of inhibitors increased due to reduced enzyme activity. This trend has been noted for *T. indica* extract for inhibition of G-sites (GSH). However, the inhibition of the catalytic efficiency of the reaction at H-sites (CDNB) does not change, whereas the apparent affinity of the substrate to the binding site is decreased as the *K*_M_ value increases. This means that the binding affinity to the enzyme is reduced, but can be resolved by increasing the concentration of the substrate.

Most polyphenolic compounds exhibit mixed (non-competitive) or uncompetitive inhibition of GSTs to GSH and CDNB substrates due to binding of the compounds to the GST binding site [56]. The substrate and inhibitor cannot bind to the enzyme at the same times in competitive inhibition, and this form of inhibition can be reversed by the use of high concentrations of substrate [57, 58]. Inhibition of human GSTP1-1 involves conformational changes as suggested by the antioxidants, tocopherols and tocotrienols. Furthermore tannin compounds have the potential to bind proteins including the GST enzyme through hydrogen bond formation causing steric hindrance and enzyme inactivation [59].

Competitive inhibition was associated with *T. indica* extract for GSTP1-1 activity is expected to avoid, or at least limit, the development of anticancer drug resistance due to its competition with it.

Cell culture has been successful in practice for centuries for ease of operation and provides a platform for exploring protocols of extended biochemistry with the possibility of regulating drug discovery and development [17].

In this study, the cytotoxicity of the most effective inhibitor of GSTP1-1 activity; *T. indica n*-butanol fraction, on the following human tumor cell lines: liver HePG 2; colon HCT116. Lung A549; breast MCF-7; PC3 prostate and normal skin fibroids, BJ1 was investigated. The results indicated that MCF-7 cell lines (72%) and HePG2 (52%) are the only ones affected by *T. indica* cytotoxicity. A close degree of cytotoxicity to methanol extract from *T. indica* seeds has been documented in the research of Hussain et al. [7] on two cancer cell lines [cancerous sarcoma (RD) and human lymphoma cell line (SR)]. Seed extracts displayed a higher inhibition rate at a concentration range of 0.1‑1000 μg/ml for both RD and SR cell lines. The findings of this analysis show that the seed extract has a good anticancer effect.

Unlike our findings, there was no obvious toxicity in Gorg et al. (2018) cell-based assays when human skin cancer (G361) and normal human lung cancer cells (TIG-3) were exposed to water or 95% ethanol of *T. indica* (range 0.01‑1.0%, at least 4 weeks) for 48 h compared to control and without any effect on the activity of cancer-promoting proteins.

In the present results, the non-affected forms of cancer cell line (colon HCT116 and lung A549) compared to the induced PC3 prostate cell line were observed. These conflicting results indicate that the effect of the compounds may differ, and further study is required for a thorough analysis of certain unknown compounds and signaling pathways that lead to tumor progression.

In the tumor microenvironment, individual tumor cells are present at different stages of proliferation, autophagy, and apoptosis. Cancer cell survival, proliferation, and metastases are affected by certain tumor microenvironmental compounds (cytokines) that interact with cells and regulate complex signaling pathways. The direct effect of these compounds on cancer cells is a controversial topic and reports of both tumor and anti-tumor effects have been reported. Anti-tumorigenic roles include inhibition of growth and activation of apoptosis. However, other studies have shown that these cytokines can promote tumor formation by inhibiting apoptosis and increasing proliferation [60].

Findings of Harris et al. [61] conducted on 54 naturally occurring phenols for assessment at physiologically appropriate concentrations for their ability to alter the viability of PC12 cells in response to serum deprivation, chemotherapy agent etoposide, and apoptogen C2-ceramide; demonstrated significant mitogenic, cytoprotective, and antiapoptotic biological activity of plant phenols in neoplastic cells at physiologically appropriate dietary concentrations that should be included in chemopreventive and chemotherapy strategies. Where, antiapoptotic and, unexpectedly, mitogenic activity was most commonly found for benzoic acid, cinnamic acid phenolic activity.

According to our results, the high concentration of protocatechuic acid was reported in the *n*-butanol extract of *T. indica* is one of the phenolic compounds found in this extract that can be linked to the anticancer effect observed in both MCF-7 and HePG2 cell lines. Protocatechuic acid is a benzoic acid derivative and is a basic polyphenol compound. This is present mainly in a variety of fruits and vegetables. It is stated to have anti-cancer, anti-inflammatory, anti-viral, anti-diabetic, and anti-atherosclerotic properties. Its function in the protection of the heart, liver, kidneys, nervous system, and reproductive organs has also been documented [62].

In the present cytotoxic test for MCF-7 cells, the IC_50_ value is 68.5 μg/ml of *n*-butanol *T. indica* fraction was observed compared to 26.1 μg/ml of doxorubicin and 1.7 μg/ml of tamoxifen, respectively.

Doxorubicin (Dox) is an anthracycline drug used to treat various types of cancers. Dox is known to act by interacting with the enzyme topoisomerase IIα, leading to an accumulation of DNA breaks and ultimately to cell death. Nevertheless, resistance to Dox is a serious problem, and a variety of interventions have been suggested such as minimize drug absorption, enable drug detoxification, increase drug flow and DNA repair capacity, and prevent apoptosis from occurring [1].

Tamoxifen is a selective estrogen receptor modulator and has been one of the key hormone therapy approaches used in the treatment of breast cancer for decades. Sadly, many patients with breast cancer have developed resistance to tamoxifen therapy along with tumor progression. Numerous molecular mechanisms for endocrine resistance have been suggested, including alteration of the signal transduction pathway for the estrogen receptor, modification of the signal that regulates the cell cycle and ability to survive, and activation of escape pathways who provide tumors with alternative reproductive and survival stimuli [33].

New strategies for reducing the required dose of anticancer drugs would be beneficial. The identification of the anticancer properties of widely used food products and other medicinal plants may therefore provide potential solutions for this reduction. Research suggests that the use of natural products and chemotherapeutic agents results in beneficial or synergistic effects on cancer cells [63]. Natural anticancer agents, like phenolic compounds, produce considerably less toxicity, are safe and readily accessible; thus, combination therapy using natural anticancer drugs should be encouraged alongside commercially approved anticancer drugs in order to reduce limitations in the treatment of advanced cancers [33].

The combination of herbs and drugs has been shown to be effective in most medical practices; however, findings from some studies have suggested adverse reactions. The majority of studies on the negative effects of herb-drug interaction come from status reports, but with little detail. The highest level of evidence for herbal interactions comes from case reports and drug trials [64].

Evidence suggests that the use of natural products and chemotherapy agents contributes to additive or synergistic effects on cancer cells. In this study, the experimental treatment of *T. indica* extract + tamoxifen eliminated almost percent of the inhibition of each individual, i.e., their cytotoxicity was canceled.

Polyphenol exercises its anti-cancer activity in certain types of cancer, attributed to their targeting of antioxidant, anti-inflammatory, and anti-estrogenic mechanisms [33]. The predominance of the natural antioxidants α-tocopherols (vitamin E) and γ-tocopherols and, in particular, the presence of δ-tocopherol in the tamarind seeds (*T.indica*) alongside compounds such as flavonoids, ascorbic acid, β-carotene, and polysaccharides [65].

The presence of vitamin E as an antioxidant can interfere with the pharmacological action of certain anticancer drugs that rely on the development of reactive oxygen species as part of their mechanism of action. Tamoxifen, an anticancer medication, has been developed for the treatment of breast and prostate cancers, both of which have their anti-proliferative and pro-apototic activities blocked by α-tocopherol (vitamin E).

Khallouki et al. [62] stated that many women seeking breast cancer treatment are likely to take antioxidant dietary supplements, which could have a detrimental effect on their clinical outcomes. For example, the intake of vitamin E during prophylactic or curative treatment can affect the clinical outcome of patients treated with tamoxifen. This example of vitamin E-tamoxifen interaction was considered to have a negative effect interaction. Another example of a negative effect is the potential induction of CYP450 isoenzymes by valerian (*Valeriana officinalis*), a sleep aid plant, which can adversely affect the effectiveness of anticancer drugs. Even so, laboratory and clinical studies research has failed to draw any logical conclusion that supports the adverse association of valerian with anticancer drugs [65]. Also, herbs containing ephedra or caffeine (cola nuts, guarana, mate, and green tea), often used in combination with cardiovascular effects applied to many herbal products for weight loss, may have the benefit of antihypertensive drugs [65].

On the other hand, a positive example of herb-drug interaction was seen in the combined effect of tamoxifen and green tea extract produced synergistic inhibition of estrogen receptor cell proliferation-positive breast cancer cells, which include MCF-7. Green tea has been shown to inhibit breast cancer growth by having a clear antiproliferative effect on cancer cells and indirect inhibiting effects on cancer-associated endothelial cells [63].

## Conclusions

This study may lead to a change in the understanding of the chemotherapy protocol for cancer patients, as the findings obtained were theoretically unexpected. The use of the plant extract (*T.indica*) with the medicinally approved drug has contributed to the complete cancelation of the effect of the drug and also to the cancelation of the effect of the herbal extract, which has been shown to be effective in killing cancer cells as shown in the results, indicated the following:

- The extract of *T. indica* has a cytotoxic effect on the MCF-7 cell line with 72% inhibition of cell viability.
- In some situations, plant products may have the opposite effect to the desired drug, which reduces the effect of the drug.
- The combination of the plant product and the drug may result in the interaction of its components with the drug or may result in a change in the pharmacodynamics of the drug.
- More studies are needed to investigate the molecular mechanisms associated with the anti-cancer activity of natural compounds and their anti-cancer drug interactions in order to convert them into effective chemotherapeutics.

## Data Availability

All data generated or analyzed during this study are included in this published article.

## Abbreviations

TPC: Total polyphenol content
TFC: Total flavonoid content
HPLC: High-performance liquid chromatography
DPPH: 1, 1 diphenyl-2-picryl-hydrazyl
HePG 2: Hepatocellular carcinoma cell line
HCT116: Colon cell line
A549: Lung carcinoma cell line
MCF-7: Breast cancer cell
PC3: Prostate cell line
BJ1: Normal skin fibroblast
